# Examining the Associations between COVID-19-Related Psychological Distress, Social Media Addiction, COVID-19-Related Burnout, and Depression among School Principals and Teachers through Structural Equation Modeling

**DOI:** 10.3390/ijerph19041951

**Published:** 2022-02-10

**Authors:** Turgut Karakose, Ramazan Yirci, Stamatis Papadakis

**Affiliations:** 1 Department of Educational Sciences, Faculty of Education, Kutahya Dumlupinar University, Kutahya 43100, Turkey; turgut.karakose@dpu.edu.tr; 2 Department of Educational Sciences, Faculty of Education, Sutcuimam University, Kahramanmaras 46050, Turkey; yirci@ksu.edu.tr; 3 Department of Education, University of Crete, 74100 Rethymno, Greece

**Keywords:** COVID-19, psychological distress, social media addiction, burnout, depression, internet addiction, principal, teachers, K-12 education, structural equation modeling

## Abstract

This study aims to investigate the relationships between COVID-19-related psychological distress, social media addiction, COVID-19-related burnout, and depression. The research, which was designed according to the relational survey model, was conducted with the participation of 332 school principals and teachers who received graduate education in the field of educational administration. Research data were collected through online surveys and then structural equation modeling (SEM) was used to test and analyze the proposed hypotheses. The study’s findings revealed that COVID-19-related psychological distress strongly predicted COVID-19-related burnout. In this context, as the psychological distress associated with COVID-19 increased, the sense of burnout associated with COVID-19 also increased. However, it was found that burnout associated with COVID-19 significantly and positively predicted depression. SEM results revealed that COVID-19-related psychological distress directly affected COVID-19-related burnout, depression, and social media addiction. In addition, it was determined that an indirect effect of COVID-19-related burnout and social media addiction exists in the relationship between COVID-19-related psychological distress and depression.

## 1. Introduction

From the initial case of the new coronavirus reported in China in December 2019, the virus spread rapidly on a global scale. The Chinese Government’s complete shutdown of all cities in the Hubei province, where the virus was first detected, in an attempt to halt the spread of the virus could not prevent its global impact [1,2]. China’s close relationship with other world countries in the field of trade and business paved the way for the virus to quickly spread to other countries. The rapid spread of COVID-19 soon created a pandemic which significantly affected most areas of human life, from small, medium and large enterprises, to national economies, health, food safety, employment, travel, the environment, energy markets, and the education sector [3].

As a result of the efforts to develop a vaccine to eliminate the coronavirus and the effects of the disease, urgently approved vaccines against COVID-19 began to be administered. However, early hopes of a fully effective vaccine were soon blighted, following the discovery of new variants of the virus, neurological diseases, and other symptoms and long-term effects reported in COVID-19 patients. In light of these emerging developments, the potential for the pandemic to reach uncontrollable levels was also talked of [4]. Much has been revealed about SARS-CoV-2 through scientific research, which has subsequently led to unprecedented scientific progress in the development of COVID-19 vaccines. However, there is still great uncertainty regarding the future course of the pandemic that concerns virologists, epidemiologists, and others in the wider science community. Although COVID-19 vaccines are now available in many countries, it does not yet seem possible to completely eradicate the global crisis caused by the pandemic [5].

As a result of the COVID-19 pandemic, people’s social lives, close relationships, and economic well-being have been adversely affected, with daily life routines having changed considerably since the start of the outbreak. Unprecedented social distancing measures were soon put into practice, which significantly restricted social life. Some radical measures were taken, such as local, regional, and international travel bans, quarantine practices, and the transition of education at all levels to distance-based mediums. The ongoing risks and effects of the pandemic triggered negative emotions in many such as fear, anxiety, and anger [6,7,8,9,10]. For this reason, it is necessary to consider the negative aspects as well as the benefits of the measures taken to prevent the spread of the virus, because uncertainty and fear threaten not only physical health but also mental health and well-being [11].

## 2. Literature Review

No definitive timetable has been determined by health authorities and national administrations as to how long the legal measures to combat COVID-19 will continue. Although various vaccines have been developed to help protect against contracting the virus and its health impact, it has been reported that there is no definitive solution to prevent the spread of the virus. Therefore, discussions on whether the pandemic may persist in the coming years are still set to continue, and the psychosocial effects of COVID-19 are also expected to cause significant impact well into the future. The mental health effects of social isolation brought about by the various quarantine measures implemented to slow the spread of the virus have not yet been sufficiently investigated. During a pandemic, the need for increased psychological as well as medical support should be taken into account, with access ensured to appropriate mental health facilities and support. While setting measures aimed at reducing the rapid spread of the virus forms the natural center of pandemic management strategies, it has also become evident that it is even more important to encourage and facilitate practices that support both the physical and psychological health of individuals [12]. In this context, there is a need for measures aimed at supporting the psychological health of education sector workers, as well as healthcare workers who are at the forefront of the fight against the pandemic. This strategy is considered important in terms of ensuring that students continue to receive school-based education [13,14] in a healthy psychological environment.

In the last 50 years, significant developments and improvements have been achieved in educational activities worldwide and at all levels. However, the COVID-19 pandemic has posed perhaps the greatest challenge that any national education system has ever faced [15]. The global pandemic has deeply shaken education systems to the core, with the impact having directly affected some 1.6 billion students in more than 200 countries. The fact that more than 94% of the world’s student population has been affected during this difficult and critical period clearly shows the extent of the pandemic’s impact on global education. The reopening of schools following the relaxation of restrictions has also brought with it certain harsh measures. During the period when schools were closed to face-to-face education, online learning tools and distance education were seen as the savior to a continuance of basic level education. However, the transition from traditional face-to-face classroom-based learning to a fully online learning environment presented a very different experience for both students and educators, and both were expected to adapt quickly to this new situation in just a very short period [16]. Along with emergency distance education, teachers were asked to utilize information technologies within online educational environments regardless of their digital literacy levels [17].

In addition to their new responsibilities as part of the online learning-teaching process, teachers are expected to take on more responsibilities with the reopening of schools. In this context, the role of teachers has expanded beyond being educators to include hygiene and infection control supervisors. In addition, even greater emphasis has been placed on taking care of the mental health and well-being of students. The increasing responsibilities of teachers during the COVID-19 pandemic have resulted in considerable emotional and psychological strain. In this context, the literature has revealed that anxiety, depression, stress, post-traumatic stress symptoms, burnout, fatigue, and sleep problems are the more common ailments reported by teachers [18,19].

When considering its scope and effect, COVID-19 should not only be seen as a new type of virus, but also as a major source of psychosocial problems. In addition, psychological factors related to how people cope with the threat of infection, fear of losing loved ones, and grieving the actual loss of loved ones can contribute to increased levels of psychological distress. Therefore, both the medium-term and long-term effects of the virus on mental health should not be underestimated [20,21,22,23]. It has been stated that the fear of losing employment or income, losing loved ones, or experiencing serious and even life threatening health problems are among the most intense negative emotions seen during the pandemic [24]. On the other hand, Răducu and Stănculescu [25] stated that teachers’ feelings of burnout are among the most common negative emotional states reported during the pandemic period.

After the first case of the virus emerged, almost all schools worldwide suspended face-to-face education, and emergency distance education tools were rapidly put into use to offer some form of continuance for their students’ education. Millions of students and teachers have tried to adapt to this new situation; however, teachers have mentioned being in significant need of in-service training and psychological support during this process [26]. The COVID-19 pandemic period was not considered easy for either teachers or their students, and students’ anxiety and stress levels reportedly increased during this troubling period [27,28,29,30].

With distance education, the use of the Internet has increased further with both lessons and follow-up sessions having moved to the online environment. Consequently, the time spent by both students and teachers in using the Internet has increased significantly. For example, in a study conducted by Baltacı et al. [31], it was revealed that university students had difficulty in controlling their Internet usage levels during the pandemic period. With a significant proportion of the study’s university student participants having spent more than 5 h a day using the Internet during the COVID-19 pandemic, this increased usage may pose greater risks in terms of problematic Internet usage levels. Magaña et al. [32] reached a similar conclusion in their research sample studied in Spain, and as a result of their research, it was determined that the duration of Internet usage by teacher candidates during the pandemic increased at a level that could be considered psychologically harmful.

During the curfews imposed to prevent the further spread of COVID-19, teachers were required to use the Internet for a wide variety of purposes, as did those employed in many different professions who spent increased amounts of time working from home. In this context, social media became one of most increasingly used Internet tools. In a study by Truzoli et al. [33], it was determined that social media networks were used more frequently by females than males. In addition, the majority of teachers reportedly also used the Internet for purposes other than online teaching, and for periods varying from between 1 and 3 h daily. It was also reported that a small number of teachers participating in the research used the Internet and social media for more than 7 h a day.

Studies have found that the problematic use of social media is significantly associated with psychological distress and stress, both from direct and indirect means [34,35]. Therefore, in order to protect human mental health during a pandemic, greater focus should be placed on reducing the excessive usage of social media, and increased levels of physical activity should be encouraged instead. For this purpose, examples of physical activity that do not require expensive equipment should be encouraged via television broadcasts, government websites, and billboard advertisement campaigns, as well as promotions aimed at encouraging users to consciously control and reduce the time spent using the Internet. The addictive use of social media has yet to be formally recognized as a scientific psychiatric case; however, scientific studies conducted in this context have revealed that social media addiction may have potentially negative consequences that should not be ignored [34].

Although it has been stated that the effects of the COVID-19 pandemic are being investigated more clinically, and that such studies are mostly focused on healthcare professionals, the number of studies examining the psychological aspect of the pandemic and its effects on school administrators and teachers from an educational perspective has been very few [20,36]. Therefore, in the current study, the relationships between the psychological distress associated with COVID-19, social media addiction, burnout, and depression associated with the pandemic among school principals and teachers are aimed to be comprehensively examined. Additionally, the study aims to reveal implications in terms of the scientific literature on this topic. The current study also extensively analyzed the relationships between certain variables within the scope of the research, and functional solutions suggested aimed at policymakers and practitioners in education within the framework of the findings.

## 3. Materials and Methods

### 3.1. Purpose of the Study and Hypotheses

The study aimed to examine the relationships between the psychological distress associated with COVID-19, social media addiction, COVID-19-related burnout, and depression amongst school principals and teachers by way of structural equation modeling (SEM). With scientific research conducted according to the framework of the modern positivist paradigm, the goal of many research studies is to develop a solid theoretical framework and then to extensively test that theory. One of the techniques frequently applied in this context is SEM, which as an approach with the flexibility to model relationships between multiple variables, and to test causal relationships between the variables [37,38]. One of the strengths of SEM is that the causal processes under investigation are represented by a set of structural equations (such as regression) and that the structural relationships can be modeled pictorially in order to provide a much clearer conceptualization of the theory being studied [39].

The following hypotheses were developed within the framework of the general purpose of the current study.

**Hypothesis 1** **(H1).** *Psychological distress associated with COVID-19 has a significant effect on COVID-19-related burnout.*

**Hypothesis 2** **(H2).** *Psychological distress associated with COVID-19 has a significant effect on depression.*

**Hypothesis 3** **(H3).** *Psychological distress associated with COVID-19 has a significant effect on social media addiction.*

**Hypothesis 4** **(H4).** *Burnout associated with COVID-19 has a significant and positive effect on depression.*

**Hypothesis 5** **(H5).** *Social media addiction has a significant and positive effect on depression.*

**Hypothesis 6** **(H6).** *There is an indirect effect of COVID-19-related burnout on the relationship between COVID-19-related psychological distress and depression.*

**Hypothesis 7** **(H7).** *Social media addiction has an indirect effect on the relationship between COVID-19-related psychological distress and depression.*

**Figure 1:**
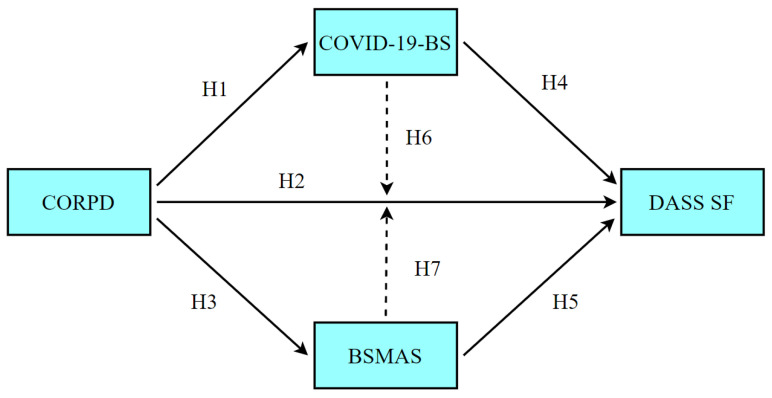
Hypothesized relationships of the research model (CORPD = COVID-19–Related Psychological Distress; COVID-19-BS = COVID-19 Burnout; BSMAS = Social Media Addiction; DASS SF = Depression).

Figure 1 illustrates the hypothetical model regarding the relationships between the variables examined in the study.

### 3.2. Study Design

Descriptive correlational design was used in the study in order to examine the relationships between COVID-19-related psychological distress, social media addiction, COVID-19-related burnout, and depression amongst school principals and teachers. The data of the research were analyzed using IBM’s SPSS (Firat University, Elazig, Turkey) and AMOS package programs (D. Nacar, Kahramanmaras, Turkey). In this context, the path analysis method was applied, which is a statistical method of analysis used in SEM. In path analysis, causal relationships between variables can be clearly identified, and is also used as an important method to investigate the direct and indirect effects of causal variables [40,41,42]. Path Analysis is similar to multiple regression analysis, but has superior and flexible features compared to multiple regression analysis. In path analysis, there can be more than one dependent variable, and variables can be both the dependent and independent. Path analysis allows for the examination of more than one regression model at the same time; hence, both indirect and direct effects can be measured at the same time [43]. Within the scope of the current study, correlation values, Cronbach’s alpha coefficients, arithmetic mean, skewness, and kurtosis values of the scales were analyzed using the SPSS package program, whilst confirmatory factor analyses of the scales, the goodness-of-fit values of the model, and testing of the hypothetical model were conducted using the AMOS program.

### 3.3. Participants

One of the strengths of SEM is that it allows for different data types to be used in examining complex relationships and offers a flexible structure for comparisons between alternative models. However, deciding what sample size should be used in SEM-based research is a challenge frequently encountered by researchers, reviewers, and authors alike [44]. Since many emotional states studied in the social sciences (e.g., happiness, depression, anxiety, cognitive, and social competence) cannot be observed directly, they are often measured by way of using multiple indicators, which are subject to measurement errors. With a sufficient number of participants (*n*), SEM enables researchers to more easily establish and reliably test hypothetical relationships between structures and their observed indicators, as well as between theoretical structures [45]. Despite this strength, SEM is also considered notorious for its sample size requirements. Structural equation models can be quite large with numerous (observed and latent) variables. Sufficient data are therefore needed in order to accurately estimate numerous parameters to an acceptable level of quality [46]. Many studies have stated that small sample sizes in SEM studies (for example, *n* < 200) may cause certain problems for the researchers [44,45]; hence, collecting of more than 200 data items in SEM-based studies is highly recommended. Bentler and Chou [47] recommend that there are at least five observations per variable (5:1) in determining the minimum sample size. However, some researchers have stated that this ratio should actually be 10:1 [48,49,50,51,52]. Considering the evaluations published in the literature, data were collected within the scope of the current study from a total of 365 participants.

The research was conducted with graduate students who were either employed as school principals or teachers in Turkey, and studying for a Master’s degree at the Kütahya Dumlupınar University’s Graduate Education Institute in the Department of Educational Administration during the 2021–2022 academic year. According to the official statistics of the institute, the total number of the teachers and school principals registered as master students is 1256 [53]. The researchers sent an invitation email to 628 randomly chosen master students explaining the purpose of the research and they were kindly required to reply the email either positive or negative. The scales were sent to the students who replied the email and accepted the invitation. According to Saunders et al.’s [54] formula, a sample of 294 participants is sufficient to represent a research population of 1256. The data were collected electronically through Google Forms, with 365 participants included in the sample through simple random sampling. As a result of the initial analysis, 33 data forms were subsequently excluded from the dataset because they included extreme values or some data elements were missing. Therefore, the analysis stage of the research was conducted with a total of 332 data items.

The sociodemographic characteristics of the participants are presented in Table 1.

When Table 1 is examined, it can be seen that the number of participants in the 20–35-year-old age range was lowest. One of the main reasons suspected for this finding is that newly recruited teachers or teachers with shorter tenures are mainly found to be working in the rural areas of Turkey. It was also determined that the social media platform most used by the participants was WhatsApp, followed by Instagram. The main reason why WhatsApp was the application used the most may be because the platform is generally preferred by schools for the sharing of school announcements, documents, and pictures, and text-based messages. Especially during the pandemic period, teachers, students, and school administrators have used this practice extensively in their communications during the education process. Thanks to user groups created using the WhatsApp platform, an effective communication network has been created between students, teachers, parents, and school administrators. YouTube, one of the most popular social media platforms, was predominantly used by the participants for video sharing. When the social media platforms used by the participants according to their age are examined, it can be seen that Facebook was preferred by the older participants, whilst Instagram was used more by those aged relatively younger.

### 3.4. Data Collection and Data Analysis

#### 3.4.1. Measurements

The data of the study were collected through four different scales, in addition to a personal information form. These scales used were the COVID-19 Related Psychological Distress Scale (CORPD), the COVID-19 Burnout Scale (COVID-19-BS), the Depression Stress and Anxiety Scale (DASS-21), and the Bergen Social Media Addiction Scale (BSMAS). However, within the scope of the current research, the 7-item “Depression Subscale” (DASS SF) was used in place of the entire DASS-21 scale. The scales used in the study were reviewed and their application approved by the Kütahya Dumlupınar University Graduate Education Institute, with legal permission granted for the study to be performed with graduate students (Permit: E-75621633-600-46907).

##### COVID-19 Related Psychological Distress Scale (CORPD)

The COVID-19 Related Psychological Distress Scale (CORPD) scale was developed by Feng et al. [55] and was subsequently adapted to the Turkish context by Ay et al. [56] The original version of the scale, which was created as a 5-point, Likert-type instrument, consisted of 14 items and two dimensions. Response options ranged from 1 = strongly disagree to 5 = strongly agree. High scores obtained from the scale indicate high levels of psychological distress. In the analyses of the Turkish adaption of the scale, two items (“If I were infected with COVID-19, I might not be able to recover from it” and “When I see someone sneeze, I suspect s/he might be infected with COVID-19”) were excluded from the CORPD Turkish form because they loaded the factor on two dimensions at the same time, had a low factor loading, or low item-total correlation (< 0.32). The Turkish version of the scale therefore consisted of 12 items. The Cronbach’s alpha coefficient was originally found to be between 0.80 and 0.88, whereas in the current research, the Cronbach’s Alpha coefficient was recalculated as being 0.93. For the current study, the validity and reliability analyses of the scale were repeated and values of the fit indices for the 12-item, two-dimensional scale were chi-square *x*^2^/*df* = 3.914, SRMR = 0.032, RMR = 0.054, CFI = 0.95, NFI = 0.93, AGFI = 0.86, and GFI = 0.92. The findings revealed that the scale’s values for fit indices were either at an acceptable fit level or at a good fit level.

##### COVID-19 Burnout Scale (COVID-19-BS)

The COVID-19 Burnout Scale (COVID-19-BS) consists of 10 items. The scale was adapted to the Turkish context by Yildirim and Solmaz [57]. In the adaptation study, the Burnout Measure-Short Version scale developed by Malach-Pines [58] was used. The COVID-19-BS scale was developed as a 5-point, Likert-type instrument (1 = never–5 = always), with a lowest possible score of 10 and a highest of 50. A high score indicates the respondent having experienced high levels of COVID-19-related burnout. As a result of exploratory and confirmatory factor analyses, it was revealed that the 10-item Turkish version of the scale had a high level of construct validity. In addition, in the adaptation study of the scale into Turkish, the Cronbach’s Alpha coefficient was found to be 0.92. Within the scope of the current study, the reliability and validity values of the COVID-19-BS scale were re-examined and the Cronbach alpha coefficient of the scale was calculated as being 0.88, whilst the fit index values of the one-dimensional structure of the scale were chi-square *x*^2^/*df* = 2.743, SRMR = 0.044, RMR = 0.035, CFI = 0.97, NFI = 0.94, AGFI = 0.87, and GFI = 0.95. These findings show that the fit indices of the scale’s construct validity can be described as being at an acceptable or good fit level.

##### Depression Scale (DASS SF)

The 42-item Depression Stress and Anxiety Scale, which was first developed by Lovibond and Lovibond [59], was later reconfirmed as the 21-item Depression Stress and Anxiety Scale (DASS-21) by Henry and Crawford [60]. In the current study, the 7-item Depression Scale (DASS SF), a subscale of the DASS-21, was employed. The DASS SF, which was adapted to the Turkish context by Yilmaz et al. [61], is presented as a 4-point, Likert-type scale coded as 0 = not suitable for me, 1 = somewhat suitable for me, 2 = usually suitable for me, and 3 = completely suitable for me. In the Turkish adaptation of the scale, the 7-item structure was confirmed and no items were removed from the scale. In Yilmaz et al.’s [61] study, the Cronbach’s Alpha coefficient was calculated as 0.87. In the current study, the reliability coefficient was re-examined and was also found to be 0.87, and validity analyses were also re-performed and were revealed to fit the index values of chi-square *x*^2^/*df* = 2.803, SRMR = 0.032, RMR = 0.015, CFI = 0.97, NFI = 0.96, AGFI = 0.94, and GFI = 0.97. These values revealed that the fit indices in the construct validity of the scale were found to be at either an acceptable or a good fit level.

##### Bergen Social Media Addiction Scale (BSMAS)

The 6-item Bergen Social Media Addiction Scale (BSMAS) was developed by Andreassen et al. [62], whilst the Turkish adaptation was performed by Demirci [63]. The scale is answered according to a 5-point, Likert-type rating (1 = very rarely–5 = very often), with a lowest possible score of 6 and a highest score of 30. As the scores obtained from the scale increase, the social media addiction level of the respondent also increases. In Demirci’s [63] adaptation study, the internal consistency of the scale was found to be 0.88. Within the scope of the current research, the reliability and validity values of the scale were re-examined. In this context, the Cronbach’s Alpha reliability coefficient of the scale was calculated as 0.77, while the fit index values of the one-dimensional scale were found to be chi-square *x*^2^/*df* = 2.302, SRMR = 0.039, RMR = 0.047, CFI = 0.95, NFI = 0.92, AGFI = 0.95, and GFI = 0.98. These findings revealed that the fit indices in the construct validity of the BSMAS were of an acceptable or good fit level.

#### 3.4.2. Data Analysis

IBM’s SPSS and AMOS software packages were used to analyze the electronically collected data. Reliability coefficients for the scales used in the study were recalculated and confirmatory factor analyses were applied. In addition, the frequency and percentage values of the demographic characteristics of the sample were examined, and correlation analysis was performed to determine the direction and degree of the relations between the variables. In the testing of the study’s hypotheses, a structural equation model was established and the relationships between the variables were subsequently analyzed. A maximum likelihood method was utilized in the AMOS program and was used in the SEM. Prior to commencing the SEM analysis, it was examined as to whether or not the data met the basic assumptions of this type of analysis. In this context, sample size, missing values, extreme values, normal distribution, and multicollinearity are recommended to be examined [50], as ignoring these assumptions in multivariate statistical analyses may cause the results to be considered biased or meaningless [64]. Diagrams were created using a Flowchart Maker & Online Diagram Software [65].

In the preliminary analysis, which started with a total of 365 data items, it was determined that there were no missing data in the dataset. Univariate outliers were calculated by looking at *z*-scores, and multivariate outliers were calculated by looking at Mahalanobis distance values. In the related literature, it has been stated that data with a *z*-score of between −3 and +3 are said to meet the conditions of being normally distributed. As such, 15 data items that did not meet the univariate normality condition were determined as extreme values and subsequently excluded from the dataset. In the context of Mahalanobis distance values, 18 data items below the value of 0.001 were determined as extreme values and were also excluded from the dataset. To determine whether or not the scale scores showed a normal distribution in the analyses carried out with the revised total of 332 data items, the skewness and kurtosis coefficients of the scores were examined. These values are expected to be between −2 and +2 [66,67], and all of the scales employed in the current study were found to have met the criterion skewness and kurtosis values.

When examining the sample size, which is another assumption used in SEM analysis, it was accepted that the dataset of 332 data items also met this assumption. Another assumption tested was multicollinearity, which is a problem that occurs as a result of variables that are shown to be highly and significantly correlated with each other, causing more variables to be studied than statistically necessary, and thereby negatively affecting the SEM. Having a relationship of 0.90 or more between the variables creates a multicollinearity problem only if the sample size is sufficient [68]. In the current study, it was determined that the correlation coefficients between the variables ranged from 0.14 to 0.55 (*p* < 0.05). Based on these data, it was determined that the variables of the current research met all the assumptions of the SEM.

## 4. Results

Prior to commencing the study’s data analysis, it was examined whether or not a multicollinearity problem existed between the variables. Multicollinearity can cause erratic estimates and erroneous variances that can negatively affect the confidence intervals and hypothesis testing. Examination of the correlation matrix may help to detect multicollinearity, but that alone it is not considered sufficient [69]. Examining the Variance Inflation Factor (VIF) and Tolerance values are among the preferred methods to examine whether or not there is multicollinearity between the variables. As a general rule, tolerance values should not be less than 0.1, and correspondingly, VIF values should not exceed 10 [70,71]. In this context, the VIF and Tolerance values of the independent variables are presented in Table 2.

As can be seen in Table 2, the VIF values of the variables within the scope of the research model were found to be <10. In addition, the tolerance values were found to be greater than 0.1. In light of these findings, it was accepted that no linearity problem existed between the variables used in the analysis of the research model. In addition, skewness and kurtosis values were examined in order to see whether or not the research data exhibited a normal distribution. Skewness and kurtosis values are expected to be between −2 and +2 for the data to be accepted as normally distributed [66,67]. The mean, standard deviation, skewness and kurtosis values of the scales used in the research are presented in Table 3. Accordingly, it can be stated that the skewness and kurtosis values of all the scales support the normal distribution of the data.

When Table 3 is examined, it can be seen that the mean score ($\bar{X}$ = 3.157) of the participants according to the COVID-19-related psychological distress scale (CORPD) is higher than the mean scores of all the other scales. This confirms that the participants experienced high levels of psychological distress and stress due to COVID-19. Furthermore, the relationship between the measurement tools used within the scope of the research was examined with Pearson correlation analysis, and the results are presented in Table 4.

When Table 4 is examined, it can be seen that all of the measurement tools used were positively and significantly related to each other (*p* < 0.001). In this context, a positive relationship exists between COVID-19-related psychological distress and COVID-19 burnout (*r* = 0.347, *p* < 0.001), a positive relationship exists between COVID-19 related psychological distress and social media addiction (*r* = 0.186, *p* < 0.001), and a positive correlation (*r* = 0.164, *p* < 0.001) exists between COVID-19-related psychological distress and depression. The two variables that were found to have a higher relationship level than other variables in the study were burnout and depression related to COVID-19. The relationship between these two variables was seen to be positive and moderate (*r* = 0.559, *p* < 0.001). Furthermore, the weakest positive relationship between the variables was found between the social media addiction and depression variables (*r* = 0.141, *p* < 0.001).

### Structural Model

In this research, a Kolmogorov-Smirnov (KS) test was also conducted for the data to confirm the normality assumption. The results can be seen in Table 5.

As a result of the analyses conducted within the scope of the research, it was determined that the sample size was sufficient, that the data were normally distributed, and that there were no linearity and multicollinearity problems. In testing the measurement models and the structural model, the covariance matrix and maximum likelihood methods were used. Prior to testing the model created within the scope of the research, confirmatory factor analyses of the measurement tools used were reconstructed with the existing dataset. The fit indices of the scales obtained from the confirmatory factor analyses are presented in Table 6.

**Figure 2:**
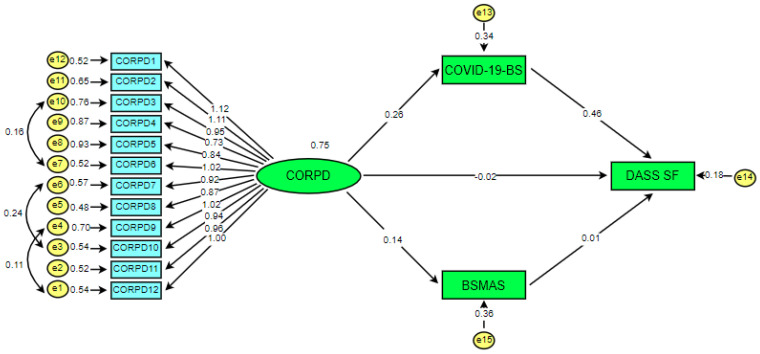
Final hypothesized model used in the research scenario.

As can be seen in Table 6, the fit indices obtained from the confirmatory factor analysis for each scale applied in the study confirm either a good level of fit or an excellent level of fit. Following this stage, the direct impact of COVID-19-related psychological distress on COVID-19-related burnout, social media addiction, and depression was investigated through SEM. In addition, the mediating effect of COVID-19-related burnout and social media addiction in the relationship between COVID-19-related psychological distress and depression was investigated. Whether or not the measurement models were validated was then analyzed with Chi-square (χ^2^/SD), GFI, AGFI, NFI, SRMR, and CFI fit indices. As a result of testing the final model, it was determined that the *t*-values of the model ranged between 3.450 and 6.563. Hair et al. [59] stated that where the *t*-value exceeds 1.96, the structural model is deemed to be significant at the 0.05 level, whereas if it exceeds the value of 2.56, it is at the 0.001 level. In the current study, the paths between the variables and the model were found to be significant at the 0.001 level. The fit indices of the hypothetical model were calculated as χ^2^/SD = 2.739; furthermore, it was determined to have the goodness-of-fit values of GFI = 0.92, AGFI = 0.88, RMR = 0.043, NFI = 0.92, CFI = 0.95, and SRMR = 0.041. These values reveal that the fit indices of the model can be considered as either good or within acceptable limits. The SEM is presented in Figure 2.

In addition, Table 7 presents the variance values, standard error, *t*, *p*, and standardized regression coefficients explained in the model regarding the direct effect of psychological distress associated with COVID-19 on other variables.

When Table 7 is examined, the standardized regression coefficient between COVID-19-related psychological distress and COVID-19-related burnout was found to be 0.268. This value shows that a positive relationship exists between psychological distress associated with COVID-19 and burnout associated with COVID-19, and that COVID-19-related psychological distress explained 36.8% of the variance of COVID-19-related burnout. According to Gignac and Szodorai [72], an effect size of around 0.10 is considered to be a “weak effect size”, whereas an effect size of around 0.20 is considered to be a “medium effect size,” and an effect size of around 0.30 is considered a “large effect size.” The study’s findings show that COVID-19-related psychological distress is a strong predictor of COVID-19-related burnout. In other words, as the psychological distress associated with COVID-19 increases in individuals, their sense of burnout associated with COVID-19 also increases. This result confirms the first hypothesis of the study (H1: Psychological distress associated with COVID-19 has a significant effect on COVID-19-related burnout).

The standardized regression coefficient between COVID-19-related psychological distress and depression was found to be 0.113. This situation reveals the existence of a weak and positive relationship between psychological distress associated with COVID-19 and depression. Psychological distress was associated with COVID-19-predicted depression at a statistically significant level. Psychological distress associated with COVID-19 explained 19.1% of the variance of depression. This result was confirmed by the second hypothesis of the study (H2: Psychological distress associated with COVID-19 has a significant effect on depression). When the standardized regression coefficient between psychological distress related to COVID-19 and social media addiction was examined, this value was found to be 0.138. This shows that psychological distress associated with COVID-19 significantly predicts social media addiction. In this context, the predictive power is close to medium. In addition, psychological distress associated with COVID-19 explained 19.6% of the variance in social media addiction. This result revealed that the third hypothesis of the study (H3: Psychological distress associated with COVID-19 has a significant effect on social media addiction) was confirmed. Finally, the findings regarding the direct and indirect relationships between the variables are presented in Table 8.

When Table 8 is examined, it can be seen that psychological distress associated with COVID-19 has a direct effect on depression, COVID-19-related burnout, and on social media addiction. When COVID-19-related burnout and social media addiction act as mediating variables, the direct effect of COVID-19-related psychological distress on depression notably disappears (β = −0.019; *t* = −0.635; *p* = 0.525 > 0.01). In the current study, the standardized regression coefficient between burnout with COVID-19 and depression was calculated as 0.459. This indicates a very strong predictive relationship between COVID-19-related burnout and depression. As the participants’ feelings of burnout due to the COVID-19 pandemic increase, their depression levels also increase. Burnout associated with COVID-19 explained 56.8% of the variance of depression. This finding revealed that the fourth hypothesis of the study (H4: Burnout associated with COVID-19 has a significant and positive effect on depression) was confirmed. When burnout and social media addiction related to COVID-19 come into play as mediating variables, the direct effect of social media addiction on depression disappears (β = 0.013; *t* = −0.337; *p* = 0.736 > 0.01). This result reveals that the fifth hypothesis of the research (H5: Social media addiction has a positive and significant effect on depression) was rejected. The use of social media as a means of entertainment and relaxation, and the fact that individuals socialized through social media during the pandemic period and maintained communication with others, albeit virtually, may have been affective in the emergence of this result. Finally, the data in Table 7 showed that the sixth and seventh hypotheses of the study were confirmed (H6: There is an indirect effect of COVID-19-related burnout on the relationship between COVID-19-related psychological distress and depression. H7: Social media addiction has an indirect effect on the relationship between COVID-19-related psychological distress and depression) (β = 0.122; CI = 0.082 − 0.167; *p* < 0.01). In other words, COVID-19-related burnout and social media addiction play a role as mediator variables in the relationship between COVID-19-related psychological distress and depression.

## 5. Discussion

The findings of the study point to a significant relationship between COVID-19-related psychological distress and COVID-19-related burnout, with participants’ sense of burnout having increased as their psychological distress increased. The so-called “new normal” working conditions that emerged following the initial stage of the pandemic, the scope of the work undertaken, and the ways in which people communicate have also changed, and this has directly affected many employees. This situation experienced during the COVID-19 period adversely affected almost all employees, regardless of their profession [73]. In this context, teachers were also faced with a significantly different working environment and approach to teaching during the early pandemic period. Alternative teaching practices were implemented across many countries, including socially distanced classrooms, hybrid teaching, or fully-online virtual teaching. However, the changing scope and increasing responsibilities of the teaching profession, the anxiety of potentially becoming infected with the virus, and anxiety based on communication with parents and students created the necessary conditions for teachers to experience increased levels of burnout and work-related stress [74,75]. In the field of education, burnout syndrome has been found to be more common amongst teachers due to the prolonged stress caused by the changes brought about by the COVID-19 pandemic [76]. However, psychological distress and burnout associated with COVID-19 have negatively affected not only teachers, but also school administrators who have a critical role in the ongoing development of educational institutions. During the initial period of the COVID-19 pandemic, the intense demands associated with the seemingly constant closing and reopening of schools and the enforcement of social distancing rules in schools exacerbated the burnout experienced by school administrators [77]. During the COVID-19 pandemic, both the communication between teachers and school administrators and also between students and their teachers continued virtually uninterrupted, although the medium of communication changed significantly in many cases. This situation also changed the concept of teachers’ working hours, requiring schools to stay in touch with stakeholders at almost every hour of every day. These and similar situations may have caused those working in the education sector to experience psychological distress and feelings of burnout.

The results of the current study revealed that a positive relationship exists between the psychological distress associated with COVID-19 and depression. School administrators and teachers have made great efforts to ensure continuity in education since the beginning of the pandemic [78]. However, sudden changes in professional practices in the field of education have caused changes in the anxiety, depression, and stress levels of education workers. The reopening of schools to face-to-face classes as the pandemic continued also impacted on the anxiety, depression, and stress levels of teachers. Silva et al. [79] determined that teachers returning to face-to-face education in schools experienced high levels of anxiety and depression. Uncertainty brought about by the pandemic, fear of contracting the virus, stress, and psychological distress caused through work provided the appropriate conditions for depression to become manifest. In addition, it is well known that while infected individuals can be psychologically affected, many uninfected individuals can also experience significant mental health issues [80]. Research by Margetić et al. [81] determined that the level of individuals who stated having experienced severe or very severe depression due to the COVID-19 pandemic was 16.9%. The results of these various studies in the literature show that the psychological experiences of individuals during the pandemic period are both multifaceted and complex.

The current study further revealed that psychological distress associated with COVID-19 significantly predicted social media addiction. Social media are Internet-based platforms where people follow current events, communicate with others, seek entertainment, and may use it as a form of escape from negative emotions such as fear and anxiety [82,83,84]. While the use of social media is seen as positive in terms of individual democracy, interaction, individual broadcasting, and a participatory culture, these technologies also attract criticism due to reasons such as hate speech, disinformation, and numerical inequality that are reported widely in social media [83]. Social media is therefore likened to a double-edged sword due to the benefits it provides and the potential for significant risk its usage also poses. It has been stated that the use of social media has a multifactorial effect on incidences of depression, anxiety, and psychological distress in individuals. Although there is evidence in the relevant literature of a relationship between the time spent using social media and depression, some research have also revealed opposing findings [85].

Researchers have emphasized that social media can be addictive because of its ever-increasing content, its continuity, and the influence it has with many users spending extraordinary amounts of time using these media [86]. Again, some research results have revealed that the time spent using social media may have an association with increased levels of anxiety, depression, and other mental health disorders [87,88]. The usage of social media has increased with the COVID-19 pandemic. On this, Gao et al. [89] stated that with the pandemic, more and more people have become dependent on the use of social media, which has increased the likelihood of encountering mental health problems. Luo et al. [90] determined that there was a 4-h increase in the use of social media per week during the pandemic period. In addition, they determined that the participants of their study experienced moderate or severe stress (10.8%), anxiety (26.4%), and depression (18.2%). These results revealed that social media addiction has had a tendency to increase during the COVID-19 pandemic period, and that a more comprehensive solution to this ever-increasing problem is required.

According to another result of the current research, when burnout and social media addiction are examined as intervening variables, the direct effect of social media addiction on depression was found to disappear. This shows that individuals used social media during the pandemic period to socialize, and therefore felt less alone. At the same time, producing content through social media, sharing posts with their target audience, and obtaining information about the course of the pandemic may have provided some element of relief to some individuals. According to Karakose et al. [91], it was determined that social media is mostly used for entertainment purposes such as watching videos, sharing photos, and that no significant relationship was detected between Facebook addiction and levels of loneliness. Yildizgorur [92] stated that social media has a wide range of uses. Since it is not possible to define such a wide-ranging environment as simply beneficial or harmful, it would be more accurate to evaluate the benefits and potential harms together. In this context, it is important to note the potentially harmful aspects of social media usage, and then to work towards minimizing such harm. Similarly, revealing the beneficial aspects of social media usage is considered important in terms of highlighting and developing these positive aspects.

Finally, the current study revealed that a direct or indirect relationship exists between COVID-19-related psychological distress, social media addiction, COVID-19-related burnout, and depression in school principals and teachers. It was revealed that teachers and school administrators, as the two key players of the education system, have seen their workload increase during the pandemic period, as well as experiencing increased psychological issues whilst adapting to the ever-changing working conditions, and that this situation has led to negative feelings, such as burnout and depression. Koyuncu and Düşkün [93] stated that workers in the education sector are going through a difficult period as they are responsible for both maintaining their own lives and protecting the rights to education of the students they are responsible for. Throughout this difficult and critical period, the COVID-19 pandemic has significantly affected people’s mental health, with one such effect being depression. It has been stated that cases of depression in individuals have increased sevenfold during the pandemic period [94]. The COVID-19 pandemic has highlighted the need to prioritize the well-being of those working in the education sector, and that this state of well-being requires the combined evaluation of cognitive, subjective, physical, and mental well-being [93]. It is important to increase efforts to improve individuals’ well-being through both the national and international agenda on public health [94,95] and to prioritize the mental health of these individuals, especially teachers and school administrators, both during and after the COVID-19 global health crisis.

### Limitations

One of the main limitations of the current research has been that the data were collected using online tools. During the period in which the data were collected, emergency distance education had ended and the summer vacation preparations had begun. Therefore, since the workload of teachers and school administrators had notably decreased at that time, the participants’ perceptions of COVID-19 psychological distress, burnout, and depression may have differed from their actual emotional state during the period when the schools were open. Another limitation of the study is that the clinical psychiatric histories of the participants were not taken into account for the depression variable. The generalizability of the results is also limited because the research sample consisted only of teachers and school principals, and only includes the opinions of participants aged between 25 and 50 years old. Despite the recent scientific reports indicating the type of personality of the respondents plays a significant role in the perception of stress and depression in the COVID-19 pandemic, the data of this research lacks the personality of the respondents. Moreover, since it was not among the aims of the research, no statistical analysis was made regarding the age variable in the research. In future studies, the relationship between the age variable and the effect of COVID-19 can be investigated in greater detail.

## 6. Conclusions

The study used structural equation modeling (SEM) to examine the relationships between the levels of psychological distress with COVID-19, burnout with COVID-19, social media addiction, and depression, which school principals and teachers experienced intensely during the pandemic period. The variables of the conceptual model created were tested through path analysis. The results revealed that psychological distress following COVID-19 directly or indirectly affects the levels of burnout, social media addiction, and depression. As a result, it was determined that the fit values of the hypothetical model tested in the study were at an acceptable level and that the model was confirmed.

In conclusion, in order to reduce the pressure felt and impact from the economic, psychological, and social effects of the pandemic on teachers and school administrators, it may be beneficial to focus on developing and delivering in-service training that aims to increase the digital literacy competencies of education workers. In this context, virtual cooperation networks aimed at increasing professional solidarity and social support within educational institutions can be created. Through this approach, the problems experienced by both teachers and school administrators may be resolved through colleague assistance, and thus the conditions that trigger psychological distress and depression due to COVID-19 can be addressed and ultimately eliminated.

## Figures and Tables

**Table 1:** Sociodemographic profile of the respondents.

Variable	Description	f (*n* = 332)	(%)
Gender	Male	156	47.0
	Female	176	53.0
Age (years)	20–35	87	26.2
	36–45	142	42.8
	46+	103	31.0
Occupation	School principal	48	14.5
	Teacher	284	85.5
Most used social media platform	WhatsApp	154	46.4
	Instagram	78	23.5
	Facebook	42	12.7
	Twitter	44	13.3
	Other	14	4.2

**Table 2:** VIF and Tolerance values of the arguments.

Scale	VIF	Tolerance
COVID-19 related Psychological Distress Scale (CORPD)	1.152	0.868
COVID-19 Burnout Scale (COVID-19-BS)	1.176	0.851
Bergen Social Media Addiction Scale (BSMAS)	1.071	0.934

**Table 3:** Mean, standard deviation, skewness, and kurtosis values of the scales.

Scale	Min	Max	$\bar{X}$	SD	Skewness	Kurtosis
CORPD	1.00	5.00	3.157	0.868	−0.541	−0.258
COVID-19-BS	1.00	5.00	2.486	0.630	0.607	0.709
BSMAS	1.00	5.00	1.924	0.609	0.837	1.752
DASS SF	0.00	3.00	0.503	0.510	1.322	1.460

**Table 4:** Correlation values between scales.

Scale	CORPD	COVID-19-BS	BSMAS	DASS SF
CORPD	1	0.347	0.186	0.164
COVID-19-BS		1	0.232	0.559
BSMAS			1	0.141
DASS SF				1

**Table 5:** Normality assumption check with Kolmogorov-Smirnov test.

Scale	Kolmogorov-Smirnov Test	Distribution
	*p*-Value	
CORPD	0.09	Normal
COVID-19-BS	0.15	Normal
BSMAS	0.21	Normal
DASS SF	0.39	Normal

Normal distribution: *p* > 0.05; Non-normal distribution *p* < 0.05.

**Table 6:** Confirmatory factor analysis results of the scales.

Scale	χ^2^/SD	GFI	AGFI	NFI	SRMR	CFI
CORPD	3.914	0.92	0.86	0.93	0.032	0.95
COVID-19-BS	2.743	0.95	0.87	0.94	0.044	0.97
BSMAS	2.302	0.98	0.95	0.92	0.039	0.95
DASS SF	2.803	0.97	0.94	0.96	0.032	0.97

**Table 7:** Values of variance explained, standard error, *t*, *p*, and standardized regression coefficients.

			Estimate	*SE*	*t*	*p*	β
COVID-19-BS	<---	CORPD	0.368	0.041	6.563	***	0.268
DASS SF	<---	CORPD	0.191	0.033	3.369	***	0.113
BSMAS	<---	CORPD	0.196	0.040	3.450	***	0.138

*** *p* < 0.001.

**Table 8:** Findings on direct and indirect relationships between variables.

	Result Variables								
	DASS SF			COVID-19-BS			BSMAS		
	β	*SE*	*p*	Β	*SE*	*p*	β	*SE*	*p*
*CORPD*	0.113	0.033	***						
$R^{2}$	0.037								
*CORPD*				0.268	0.041	***			
$R^{2}$				0.136					
*CORPD*							0.138	0.040	***
$R^{2}$							0.038		
*CORPD*	−0.019	0.031	0.525						
COVID-19-BS	0.459	0.040	***						
BSMAS	0.013	0.039	0.736						
$R^{2}$	0.311								
Indirect effect	0.122 * (0.082–0.167)								

*p < 0.01. *** *p* < 0.001.

## Data Availability

The data presented in this study are available on request from the corresponding author.
